# The Lifestyle Information and Intervention Preferences of Teenage and Young Adult Cancer Survivors: A Qualitative Study

**DOI:** 10.1097/NCC.0000000000000508

**Published:** 2017-06-15

**Authors:** Gemma Pugh, Rachael Hough, Helen Gravestock, Jessica B. Haddrell, Rebecca J. Beeken, Abigail Fisher

**Affiliations:** Author Affiliations: Department of Behavioural Science and Health, University College London (Ms Pugh, Ms Haddrell, Dr Fisher, and Dr Beeken); Children and Young People’s Cancer Service, National Health Services Foundation Trust, University College Hospital (Dr Hough); CLIC Sargent (Ms Gravestock), London, United Kingdom.

**Keywords:** Adolescent, Behavior change, Cancer, Lifestyle, Support, Young adult

## Abstract

**Background:**

Little is currently known about how best to promote healthy lifestyle choices among teenage and young adult (TYA) cancer survivors. Such data gathered from a patient-centered perspective are instrumental for the development of health behavior change interventions for young people with cancer.

**Objective:**

This study aimed to explore the lifestyle information needs of TYA cancer survivors and their preferences regarding lifestyle information and intervention delivery. Lifestyle behaviors of interest were physical activity, diet, smoking, alcohol consumption, and sun safety.

**Methods:**

A total of 13 TYA cancer survivors (mean age, 22.9 years) participated in 10 individual interviews and 1 focus group (n = 3). Each interview and focus group followed the same semistructured interview guide, which was designed to explore young peoples’ motivation behind leading a healthy lifestyle, their past experience of searching for lifestyle information, and their preferences relating to lifestyle information delivery.

**Results:**

Three core themes emerged: cancer as a catalyst to lifestyle behavior change, factors influencing health behavior change, and health behavior information preferences. Social support emerged as facilitator of both health behavior change and self-efficacy.

**Conclusion:**

Young people with cancer want age-appropriate lifestyle information on a range of topics delivered in multiple formats at various time points.

**Implications for Practice:**

Health professionals working with TYA cancer survivors should address young peoples’ lifestyle information needs throughout the cancer care pathway and support young people to foster the confidence to make, and sustain, positive lifestyle behavior changes.

## Abstract 2

Supplemental digital content is available in the text.

The improvements in teenage and young adult (TYA) cancer survival rates over the past 20 years has resulted in a growing number of young people living with and beyond the disease.^1^ However, estimated age-specific cumulative prevalence data indicate that by the age of 45 years, 95.5% of young people who have had a cancer diagnosis will have a chronic health condition of some kind, and 80.5% will have a chronic health condition defined as life-threatening.^2^ Many of the long-term consequences of cancer (eg, cardiovascular disease, metabolic syndrome, chronic fatigue, and psychosocial difficulties) experienced by TYA cancer survivors manifest as a direct result of treatment or indirectly through treatment-related adverse effects such as weight gain.^3^ Healthy lifestyle choices such as being active, eating a balanced diet, abstaining from smoking, avoiding excessive alcohol consumption, and being “sun smart” have the potential to partially ameliorate TYA survivors’ risk of chronic disease, cancer recurrence, and poor health-related quality of life.^4^–^6^ Conversely, low levels of physical activity and high levels of alcohol consumption and smoking have been associated with adverse health outcomes among TYA cancer survivors.^7^

As a result, the promotion of a healthy lifestyle is increasingly being recognized as an important aspect of care for young people affected by cancer.^8^ Moreover, previous studies have found high levels of patient-reported interest in receiving lifestyle information. One survey exploring the follow-up care preferences of childhood cancer survivors in the United Kingdom (N = 112, aged 18–45 years) found that more than half had a desire to discuss current health behaviors during a late-effect clinic appointment.^9^ Moreover, survey data from 74 TYA cancer survivors (mean age, 23 years) in the United States indicated that 85% of young people would have liked information about exercise at some point after their cancer treatment.^10^ However, despite the high level of expressed patient interest in receiving lifestyle information, TYA cancer survivors’ lifestyle behaviors are generally poor. Very few young people with cancer meet diet or physical activity recommendations, and rates of binge drinking among young people with cancer have been found tobe comparable to age-matched peers.^11^,^12^ Specifically, 1 study exploring changes in TYA cancer patients (N=98, mean age=17.3 years) physical activity across the cancer continuum (pre-treatment, during treatment, and post-treatment) found physical activity levels decline significantly during treatment and nearly a quarter of young people remain inactive post-treatment despite being active pre-treatment.^13^ Efforts are required to support TYA cancer survivors to make, and sustain, healthy lifestyle choices both during and after treatment.

At present, very little is understood about how best to deliver lifestyle information and behavior change support to TYA cancer survivors as much of the qualitative and quantitative evidence concerning the information needs and preferences of TYA cancer survivors has focused on late-effect management and psychosocial issues such as fertility, education, and relationships.^8^,^14^–^16^ Ascertaining TYA cancer survivors’ specific needs and preferences regarding lifestyle information delivery is an important step in the development and design of health behavior interventions for young people with cancer. Such data gathered from a patient-centered perspective and used in the development of lifestyle intervention programs increases the likelihood that young people with cancer will engage with the lifestyle information being provided.^17^

However, lifestyle information provision is passive and unlikely to prompt behavior change without the incorporation of behavior change techniques or an understanding of the underlying motives behind lifestyle change.^18^ Identifying barriers and facilitators of health behavior change among TYA cancer survivors is central to the design and development of interventions for this age group. Therefore, the aim of this study was to explore the lifestyle information needs of TYA cancer survivors and their preferences regarding health behavior change intervention delivery. In addition, this study aimed to explore barriers and facilitators to health behavior change among young people affected by cancer.

## Method

### Participants, Recruitment, and Ethical Approval

The participants were TYA cancer survivors aged 13 to 25 years. Consistent with the National Cancer Institute definition of cancer survivor, any young person who received a diagnosis of cancer at any point within their lifetime was eligible to participate.^19^ The participants were recruited as part of a large-scale health and lifestyle survey being delivered to young people with cancer through University College Hospital, London, and CLIC Sargent (a UK-based cancer charity supporting young people and their families). Surveys were anonymous, but the participants who completed them had the option to express interest in being involved in a qualitative study focusing on the development of a lifestyle intervention for TYA cancer survivors. Young people had the option to choose the method of participation most convenient to them (a focus group or telephone interview). It was hoped that by proposing a combination of participation methods there would be increased interest. If a young person indicated interest in taking part in either a focus group or interview, they were sent an information sheet and consent form for the current study. All interviews and focus groups were conducted by the same person (G.P.) between July 2015 and January 2016. All participants provided informed written consent before the commencement of the interview or focus group. Young people received a £15 online shopping voucher for their participation. Ethical approval for this study was provided by University College London Research Ethics Committee (reference, 6206/001) and London Hampstead Research Ethics Committee (reference, 15/LO/0764).

### Interview Topic Guide

The interviews and focus group followed the same semistructured interview guide (provided as Document, Supplement Digital Content 1, http://links.lww.com/CN/A11), which focused on 3 main themes: (i) what a healthy lifestyle means; (ii) past experiences of receiving, asking, and searching for lifestyle advice; and (iii) preferences relating to lifestyle information delivery with regard to content, format, and delivery. The participants were prompted when necessary and encouraged to share their thoughts and experiences openly.

### Analysis

The qualitative data were transcribed verbatim and checked for accuracy during the familiarization phase of the 6-phase process of thematic analysis as outlined by Braun and Clarke.^20^ After the generation of the initial list of emerging themes, several meetings between the study team (G.P., A.F., and R.B.) were held until a single list of codes and themes was agreed upon. Each core theme and any unrelated subthemes were then discussed with a TYA cancer expert (R.H.) for affirmation and identification of missing themes. An independent researcher (J.H.) then coded 3 of the interview transcripts to ensure that each theme worked in relation to each coded extract. Any disagreements were resolved through discussion. All qualitative data analyses were carried out in NVivo, qualitative data analysis software, version 11 (QSR International Pty Ltd, Melbourne, Australia). To ensure transparency, the final report and generation of results were guided by the consolidated criteria for reporting qualitative research.^21^ Percentage agreement and Cohen *κ* were calculated to determine the interrater reliability of the analysis.

## Results

From the original survey study (N = 294), 93 young people left their contact details, indicating interest in participating within this subsequent study. Of these, 13 young people responded to 1 of the 3 recruitment e-mails sent inviting them to take part in this study. In total, 10 young people took part in a telephone interview. Interviews typically lasted 30 minutes in duration (range, 19 minutes and 41 seconds to 43 minutes and 16 seconds). A single focus group (n = 3) was conducted in partnership with CLIC Sargent (project cofunders) with members of the charities’ young people’s reference group using existing nonclinical meeting facilities within the charities’ head office in London. The focus group lasted approximately 1.5 hours and was arranged at a time convenient to the group members.

The mean age of the sample was 22.9 years (range, 17–25 years), and most participants were female (n = 9, 70%). The mean age at diagnosis was 18.6 years. One participant was a TYA cancer survivor of a cancer diagnosed during childhood. Most participants (n = 6, 46%) had received a diagnosis of a hematological malignancy such as a leukemia or lymphoma (Table 1).

**Table 1:**
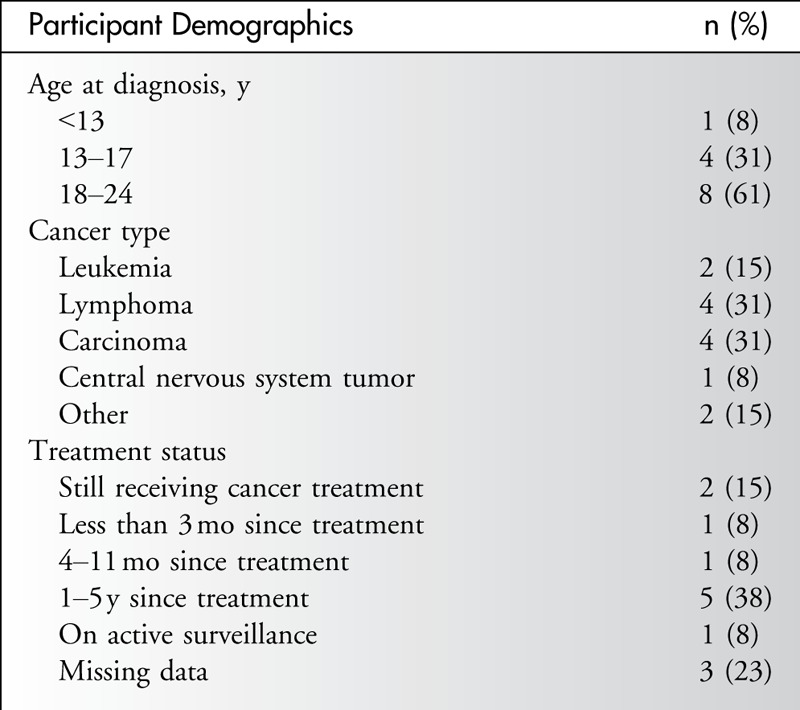
Participant Characteristics

Three core themes emerged from the interviews and the single focus group: cancer as a catalyst to behavior change, factors influencing health behavior change, and health behavior information preferences. The Figure provides an overview of the thematic map generated within this study. The interrater agreement on emerging themes was high (mean weighted percentage agreement, 99.14%; mean weighted *κ*, 0.89).

**Figure:**
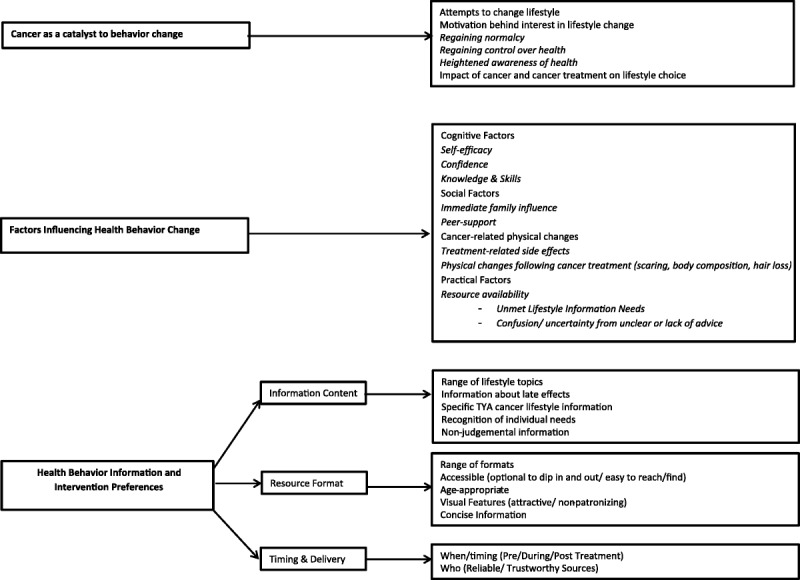
Thematic map of the lifestyle information and intervention preferences of teenage and young adult cancer survivors.

### Cancer as a Catalyst to Lifestyle Behavior Change

Young people indicated that their cancer diagnosis was often the underlying reason behind interest in lifestyle information and the catalyst behind any attempts to lead a healthier lifestyle. Leading a healthy lifestyle was seen by participants as a positive way of feeling better within themselves, regaining normality, regaining control of their health, and managing treatment-related adverse effects of cancer treatment including cancer recurrence. The participants also voiced an increased awareness of the importance of lifestyle for long-term health. This heightened awareness of health was often attributed to the effect of their cancer diagnosis on life outlook.

I think being diagnosed with cancer, you tend to be a bit more aware afterwards because you sort of don’t take your health for granted as much, I think. (Female; 17 years; age at diagnosis, 17 years; Wilms tumor)

With reference to smoking and tanning, young people often indicated aversion to smoking *“cause obviously it causes cancer”* and increased awareness of the risks of tanning and sun exposure. Often, young people indicated that their cancer and their treatment led them to be more conscious of their alcohol consumption, but some admitted being *“probably what you’d call a binge drinker.”* Two participants specifically indicated that they drink less now after their cancer diagnosis because they considered *“more important things in life”* and *“life too short to be unhealthy.”* Young people typically did not bring risk behaviors (smoking, drinking, or tanning) up within discussion unless prompted.

### Factors Influencing Health Behavior Change

It was evident that cognitive factors such as health beliefs, self-efficacy, and confidence often contributed to young people’s lifestyle behavior and engagement with health and lifestyle information.

Sometimes, you have low self-esteem and you don’t want to… It’s not that the information isn’t engaging, it’s more how you feel in yourself. (Female; 24 years; age at diagnosis, 21 years; Hodgkin lymphoma)

Peer and social support were found to be the main facilitators of confidence and self-efficacy among young people. Young people often talked about the influence that their immediate family and social network had on their lifestyle both during and after treatment and emphasized the need for peer support to come from other young people of a similar age and position to them. Many young people looked to identify with other TYA cancer survivors and described wanting to know more about their lifestyles and what was “normal” for them.

You just kind of think, well, they understand, um, and I think it’s a lot easier to get the information into your head when it’s another person’s story. Like, telling it rather than just someone whose job it is to write the information, it’s coming from someone who’s been through a similar situation. (Male; 23 years; age at diagnosis, 21 years; Hodgkin lymphoma)

All participants viewed education on the benefits of a healthy lifestyle and guidance on making lifestyle changes as important. Young people often reported that goals and progress monitoring would be useful motivational tools to encourage young people to sustain positive health behavior changes. The participants generally supported the notion that any changes should be made gradually to sustain health behavior change over time, and young people often acknowledged that a healthy lifestyle could be of benefit to everyone.

I think a lot of people, yeah, they’re disillusioned and think they can’t exercise at all. But even a little bit is good for you. So it’s just kind of making the best of what you’ve got and being able to make a bit of time to keep active. (Male; 23 years; age at diagnosis, 21 years; Hodgkin lymphoma)

Despite the high level of interest in leading a healthy lifestyle, many young people described challenges that they had faced in attempts to change their health behavior. Young people talked about the difficulty of having to navigate a “*new body*” posttreatment that is “*not the same as what it was pretreatment.*” Often, treatment-related adverse effects or late effects were the main barrier to improving lifestyle behaviors, specifically diet- or exercise-related behaviors. Young people were often frustrated by treatment-related fluctuations in weight and failed attempts to maintain or reach a healthy body weight. Dietary habits and food preferences formed during treatment were often named as one of the biggest challenges to changing diet-related behaviors.

I ate so much junk when I was on steroids that I just… it just became a habit, and I never broke it, really. So it’s taken, like, 6, 7 years for me to break the unhealthy eating I was doing for 2.5 years. (Female; 25 years; age at diagnosis, 15 years; acute lymphoblastic leukemia)

Specifically regarding physical activity, many participants shared that cancer and its associated treatment had led to a loss of confidence in their ability to exercise. One participant explained feeling self-conscious around other young people in public places such as local gyms because of cancer-related physical changes in his appearance such as “hair loss” and “a fatter face ‘cause of steroids.” In addition, young people often viewed themselves as different to other young people who had not had cancer.

I’d never dreamt of going to the gym with… I don’t mean to say ordinary people, but people of good health because I would have felt like such an outsider. (Female; 24 years; age at diagnosis, 17 years; Hodgkin lymphoma)

Geographical, financial, and time-related barriers to leading a healthy lifestyle were also mentioned by young people who indicated that traveling to support groups, paying for gyms, and preparing or cooking healthy meals were barriers to being active and eating healthily. Barriers to reducing alcohol consumption, quitting or abstaining from smoking, or being safe in the sun were often not discussed unless prompted. When prompted, young people were typically uncertain about the need to change these aspects of their lifestyle and cited lack of clear information as the primary barrier to change.

Although some young people did have a positive experience, most participants within this study reported a high level of dissatisfaction with the practical support and lifestyle information that they had received in the past. Young people typically reported previously receiving very brief information about lifestyle from health professionals; often, this advice was given within the context of treatment or during discussions about management of late effects. The participants often reported that health professionals were not forthcoming with information about lifestyle and that either no information was given or that the information they were given was vague and out of context. One young person discussed an incident when a health professional was caught off guard by their request for specific information about sun safety.

I did ask the doctor about, you know, “Was it okay to be in the sun?” I think it was a question that they weren’t really… I don’t know if he’d been that, sort of, prepared for the question. He kind of was like, […] Oh, you’ll be okay [yeah] without sun cream if you’re outside for just a little while, which didn’t really kind of answer… I’d quite like to have known, you know, what I… How… You know, can I go… Can I sit in the sun in the summer, for instance? (Female; 22 years; age at diagnosis, 21 years; HLH/chronic active EB virus)

Lack of information from health professionals often prompted young people to search elsewhere for information, most commonly online.

So the first thing I done was went on the Internet, Googling it. (Female; 24 years; age at diagnosis, 17 years; Hodgkin lymphoma)

Young people’s level of satisfaction with the information that they had found themselves varied. Some reported finding useful recipes and blogs from other cancer survivors, whereas others reported struggling to find any age-appropriate resources or any information relevant to their needs. Young people had strong views that there was not enough practical support available for young people with cancer that focused on TYA-specific issues, specifically alcohol consumption:

When you finished treatment you sort of feel a bit… not abandoned, but you are sort of on your own dealing with it yourself, trying to manage. You know, you feel like you are in charge of your own health again, which can be a bit of a responsibility. It would nice to be able to have someone … giving you a bit of guidance. (Female; 24 years; age at diagnosis, 21 years; bowel cancer)

Yeah, because, um, I would like to know, you know, can I drink? Will it affect me? You know, does it relate to, like, what kind of cancer I had? Is too much … having too much bad for me? Probably anyway. But just, just in general like if, if I wanted to go out with my friends, would I have a drink, because I don’t, I don’t know, I don’t know, if that makes sense, I don’t really know if I’m allowed to or not. (Female; 24 years; age at diagnosis 18 years; central nervous system tumor)

### Health Behavior Information Preferences

#### INFORMATION CONTENT

Young people were interested in lifestyle information on a range of topics and suggested such information should be integrated with other information topics such as late-effect management and maintaining normalcy. Interest in specific information on weight maintenance was also common. Many participants expressed a specific interest in information about socializing, particularly drinking. The participants highlighted strongly that the content of lifestyle information for TYA cancer survivors should be relevant to the needs of young people with cancer and reflect the individual needs of survivors of specific cancers (Table 2).

**Table 2:**
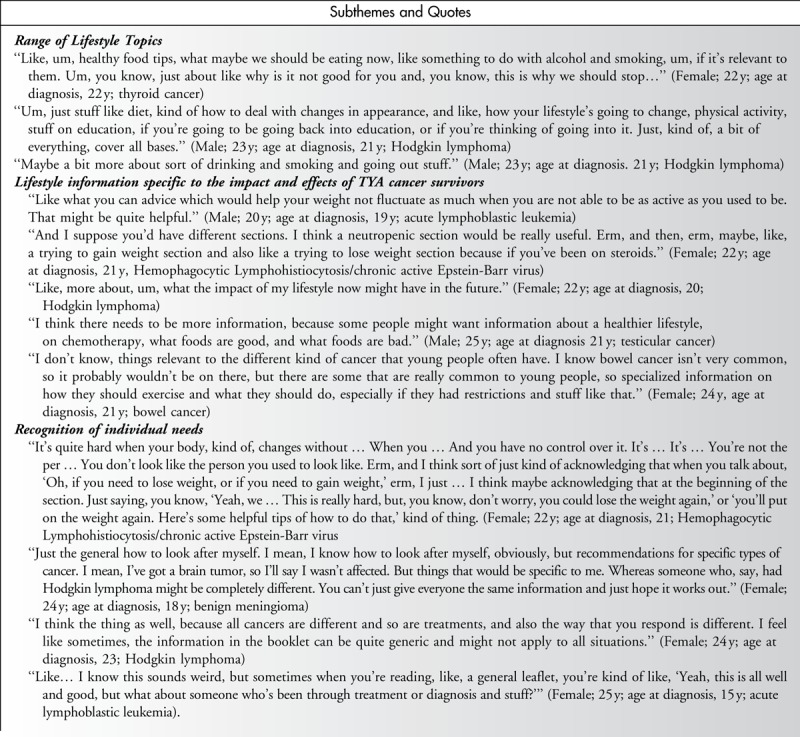
Lifestyle Intervention Preferences: Content

#### RESOURCE FORMAT

Overall, young people were interested in information presented in a variety of formats that could be accessed depending on their needs at any given time. Information delivered online or via mobile applications appealed to TYA cancer survivors because these formats were perceived as more accessible and appealing to young people (Table 3). However, several young people acknowledged that online information is only seen if it is being actively searched for. In such instances, young people highlighted the need for information to be available in multiple formats including in the form of counseling from health professionals.

**Table 3:**
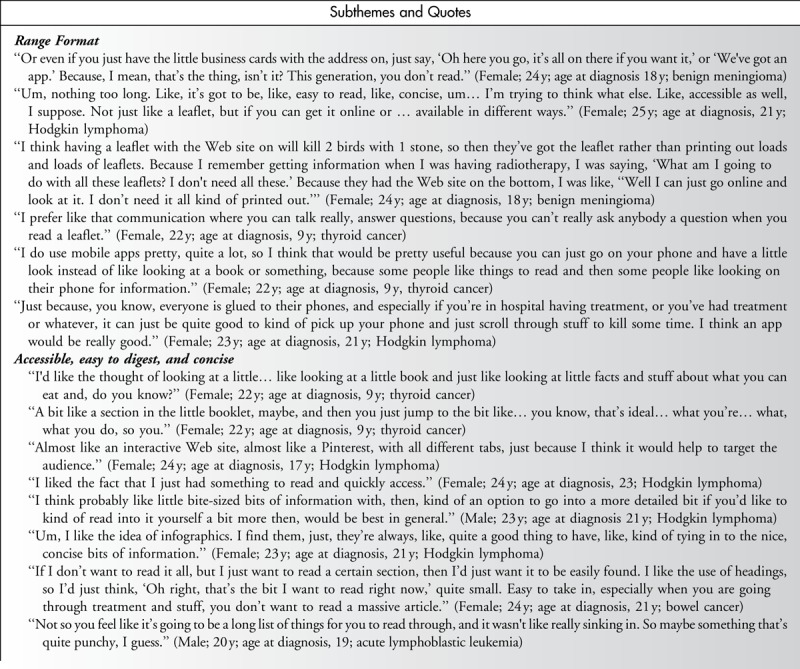
Lifestyle Intervention Preferences: Format

I think online it tends to be … you only see it then if you go looking for it…. Like, when I was diagnosed, my CLIC Sargent social worker came in, and she just gave me loads of leaflets and just said, “Look through these,” and then I looked through them, whereas I wouldn’t have gone onto a Web site and looked at it myself, really. (Male; 20 years; age at diagnosis, 19 years; acute lymphoblastic leukemia)

The participants during discussion regarding the design and presentation of lifestyle information indicated that information should be concise, accessible, attractive, and age-appropriate. Young people wanted lifestyle information that was easy to find and easy to navigate. Specific features such as designated topic sections, lists, and interactive online features such as hyperlinked content and the ability to “pin” or “like” favorites appealed.

Young people expressed frustration at having to sift through “*endless*” information resources in the past. Short articles or information presented in “*chunks that you can read one by one*” were preferred. The participants also emphasized the need for lifestyle information resources to be designed specifically for “*young people rather than children*.” Many of the participants emphasized the need for making sure both the content and format of health and lifestyle information were neither patronizing nor “*sugar-coated*.” Visual features such as use of color, infographics, and imaginative page layout were suggested by young people as being key aspects of lifestyle information design and format.

#### TIMING AND DELIVERY

No consensus of when the best time to provide lifestyle information to young people with cancer was reached. Often, young people felt that the correct time for lifestyle information delivery was dependent on an individual’s frame of mind concerning the diagnosis, treatment status, and prognosis and that such information may be best delivered as and when a person is “*ready*.” Young people were often acutely aware of the risk-taking mind-set of young people and acknowledged that information delivered at the wrong time can sometimes fall on deaf ears. The participants stressed the importance of not being overwhelmed with information and that they were more receptive to information if advice was offered in a supportive and open manner. Young people also indicated that access to the information resources should be volitional and that young people should have the opportunity to be sign-posted to relevant information to take in during their own time.

If somebody’s kind of lectured you about it, and if you’re kind of being told, ‘Oh yeah, go check this out, go check this out, go check this out,’ sometimes you just kind of think, ‘Oh, well, I don’t really want to look at that.’ So I think, if they could be given a resource pack, maybe, to go and look into themselves. (Male; 23 years; age at diagnosis, 21 years; Hodgkin lymphoma)

Throughout all interviews and focus groups, young people indicated a strong preference for information that was “*sensible*” and “*legitimate*” from reliable and trustworthy sources. Young people commonly cited well-known TYA cancer charities in the United Kingdom as the best sources of information and that they would prefer information endorsed by health professionals specializing in TYA care who “*know what they are talking about*.”

### Discussion

There is a need to provide young people who have had cancer with lifestyle information and health behavior change interventions to ameliorate their risks of chronic disease and cancer recurrence.^22^,^23^ Our findings highlight the need for readily available age-appropriate lifestyle information covering a wide range of health topics for TYA cancer survivors. Such information should be incorporated into health behavior change interventions that support young people with cancer to make and sustain positive lifestyle changes.

In this study, young people often described their cancer diagnosis as the primary reason behind their interest in lifestyle information and engagement in health behavior change. Many young people reported an increased perception of personal health risk after their cancer diagnosis and viewed adapting healthy lifestyle behaviors as a positive strategy to improve their health and well-being. There is potential that a cancer diagnosis may trigger an effective re-evaluation of health status among TYA cancer survivors and may potentially prime young people toward making healthier lifestyle choices. However, as with adult cancer survivors, it is unlikely that such spontaneous behavior change will occur, or be sustained over time, without intervention.^24^,^25^ Lifestyle information and behavior change delivery strategies should capitalize on the potential “teachable moment” when young people’s motivation to receive and act on information or behavior change support is high.

Despite the high level of engagement with lifestyle-related topics, young people in this study often described numerous barriers to health behavior change. Specifically, many young people reported that cancer-related physical changes had negatively affected their confidence and self-efficacy toward being active. This is consistent with previous reports indicating that the greatest psychosocial challenge faced by young people who have had cancer is the adjustment to physical and mental limitations resulting from their diagnosis and treatment.^14^ Content analysis of messages posted on an online forum for TYA cancer survivors confirm our findings that treatment-related physical changes or physiological problems (such as gastrointestinal issues) promote anxiety and diminish self-efficacy among young people with cancer.^26^

Conversely, social support emerged as a facilitator of both health behavior change and self-efficacy. Specifically, young people discussed the importance of social comparison and the value of knowing about the challenges that other young people with a cancer faced when making lifestyle changes. Such findings support the overwhelming evidence concerning TYA cancer survivors’ need for social support both during and after treatment and reflect the importance of behavioral modeling among adolescents and young adults.

Within this study, young people’s account of their experience and satisfaction of receiving and searching for information on physical activity, diet, drinking, smoking, and sun safety was often negative, perhaps reflecting the lack of lifestyle information or behavior change resources available for TYA cancer survivors. This is concerning given the strong correlation between unmet information needs and poor health-related quality of life among TYA cancer survivors.^27^,^28^ Young people with cancer have previously reported lifestyle information as being “overlooked” and specifically raised concerns about the effect of health professionals failing to address the consequences of alcohol consumption during treatment.^29^ A recent survey of TYA cancer survivors (N = 216; mean age, 20 years) in the United Kingdom found that the proportion of young people expressing interest in receiving advice on physical activity, diet, and weight management was greater than the proportion of young people who reported that they had actually received advice on these topics suggesting some TYA cancer survivors have unmet information needs in these areas.^30^ Addressing the specific lifestyle information needs of young people with cancer is important because information provision is a core aspect of supportive cancer care.^31^

Young people in this study were interested in age-appropriate lifestyle information specific to their needs as TYA cancer survivors. A desire for lifestyle information that includes reference to adverse-effect or late-effect management was also common. This is reflective of previous reports detailing young people’s high level of interest in discussing health behavior during late-effect consultations.^9^ In addition, consistent with previous research exploring intervention design and delivery preferences, TYA cancer survivors within this study specified that lifestyle information and health behavior change interventions should be readily available and continually accessible.^32^,^33^ Young people in this study also specified that lifestyle information should be available in multiple formats to suit individual preferences. The participants discussed the explicit advantages of lifestyle information available online, highlighting the accessibility of this format of information and intervention delivery. However, despite young people indicating a strong preference for lifestyle information and health behavior interventions to be made available and delivered online, young people acknowledged that information delivered via such mediums lack the personal support from health professionals, immediate family, and social networks, which they like. This is reflective of parent and professional opinion expressed within previous research exploring the user requirements and considerations of Web-based self-management for TYA cancer survivors: parents and health professionals both stressed the importance of online information resources not replacing face-to-face consultations and interactions with either peers or health professionals.^34^

No consensus as to when the most appropriate time to introduce lifestyle information to young people with cancer was reached. Consistent with the thoughts of TYA cancer specialists, the participants within this study typically felt that the timing and delivery of lifestyle information should take into account the individual needs of the young person being given advice. However, it was generally agreed that although health behavior change may not occur immediately it would be beneficial to introduce TYA cancer survivors to the concept of lifestyle change as early as possible in the cancer care pathway. Such early introductions to lifestyle and health behavior change information may prevent the accumulation of bad habits, trigger behavior change among some young people, and prime those who are not yet ready for lifestyle change to at least be thinking about this issue.

This study has a number of strengths and limitations. A major strength of this study is the inclusion of young people’s perspectives on drinking, smoking, and sun safety. The relevance of these behaviors to TYA cancer survivors is often overlooked within studies, which typically address diet and physical activity. Moreover, this study provides qualitative insight into some of the previously reported correlates of health behavior among TYA cancer survivors.^35^,^36^ Such information is invaluable to the development and design of health behavior interventions for TYA cancer survivors. We aimed to obtain a breadth of opinions from a wide range of TYA cancer survivors; however, very few young people answered our call for participants despite having indicated an interest in taking part during a previous study. A mixed method qualitative approach was taken, and incentives were introduced to this study to overcome these recruitment barriers; these decisions were based on previous reports detailing the difficulties typically faced within TYA cancer survivorship research.^37^,^38^ Although there were no differences between the qualitative data generated from the interviews and focus group, social barriers during the focus group may have reduced the likelihood of an individual sharing an experience or idea. It would also be reasonable to suggest that the young people who took part within this study were engaged with health and lifestyle information, and as a result, the possibility of response bias is high. Further research to clarify the lifestyle information needs and health behavior interventions preferences of TYA cancer survivors with low levels of engagement is required.

### Implications for Practice

Our findings highlight the need for readily available age-appropriate lifestyle information for young people with cancer covering a wide range of health topics. This is supported by recent quantitative data indicating that 71% of TYA cancer survivors would take up the offer of lifestyle behavior information and support if given the opportunity.^30^ Given the correlation between health beliefs and behavioral intention among TYA cancer survivors, health behavior change interventions developed and designed specifically for TYA cancer survivors should facilitate self-efficacy through social support, goal setting, and behavior tracking. Nurses and health professionals working with TYA cancer survivors should address young people’s lifestyle information needs throughout the cancer care pathway and support young people to foster the confidence to make, and sustain, positive lifestyle behavior changes. Within this context, peer-to-peer support from another young person with cancer may also be hugely beneficial to TYA cancer survivors struggling to lead a healthy lifestyle after their cancer diagnosis. Greater insight into specific correlates of health behavior and the differences between health protective behaviors such as physical activity and diet and risk behaviors such as drinking, smoking, and tanning are required. The development and design of health behavior interventions for TYA cancer survivors must also consider the perspective of health professionals working within this field.

### Conclusion

The findings from this study demonstrate that lifestyle information needs of TYA cancer survivors are currently unmet and that tailored approaches to health behavior change among this group of cancer survivors are desired. It is evident that health behaviour promotion among TYA cancer survivors is complex and lifestyle information regarding physical activity, diet, alcohol consumption, smoking, and sun safety must consider the disease-specific barriers that young people with cancer encounter when making lifestyle-related changes.

## Supplementary Material

SUPPLEMENTARY MATERIAL
