# Supplementary material for: The Lifestyle Information and Intervention Preferences of Teenage and Young Adult Cancer Survivors: A Qualitative Study
Source: Cancer Nurs. 2017 Jun 15;41(5):389–98. doi: 10.1097/NCC.0000000000000508 (PMC6259678; doi:10.1097/NCC.0000000000000508)
Supplement: SUPPLEMENTARY MATERIAL [file ncc-41-389-s001.docx]

**The lifestyle information and intervention preferences of teenage and young adult cancer survivors: a qualitative study**

The improvements in teenage and young adult (TYA) cancer survival rates over the past 20 years has resulted in a growing number of young people living with and beyond the disease.[^1^](#_ENREF_1) However, estimated age-specific cumulative prevalence data indicate that by the age of 45, 95.5% of young people who have had a cancer diagnosis will have a chronic health condition of some kind, and 80.5% will have a chronic health condition defined as life-threatening.[^2^](#_ENREF_2) Many of the long term consequences of cancer (e.g. cardiovascular disease, metabolic syndrome, chronic fatigue and psychosocial difficulties) experienced by TYA cancer survivors manifest as a direct result of treatment or indirectly through treatment related side-effects such as weight gain.[^3^](#_ENREF_3) Healthy lifestyle choices such as being active, eating a balanced diet, abstaining from smoking, avoiding excessive alcohol consumption and being ‘sun smart’ have the potential to partially ameliorate TYA survivors’ risk of chronic disease, cancer recurrence, and poor health-related quality of life.[^4-6^](#_ENREF_4) Conversely, low levels of physical activity, high levels of alcohol consumption and smoking have been associated with adverse health outcomes among TYA cancer survivors.[^7^](#_ENREF_7)

As a result, the promotion of a healthy lifestyle is increasingly being recognized as an important aspect of care for young people affected by cancer.[^8^](#_ENREF_8) Moreover, previous studies have found high levels of patient-reported interest in receiving lifestyle information. One survey exploring the follow-up care preferences of childhood cancer survivors in the United Kingdom (n=112, aged 18-45 years) found that more than half had a desire to discuss current health behaviors during a late-effects clinic appointment[^9^](#_ENREF_9) while survey data from 74 TYA cancer survivors (mean age: 23 years) in the United States indicated that 85% of young people would have liked information about exercise at some point following their cancer treatment.[^10^](#_ENREF_10) However, despite the high level of expressed patient interest in receiving lifestyle information, TYA cancer survivors’ lifestyle behaviors are generally poor. Very few young people with cancer meet diet or physical activity recommendations and rates of binge drinking among young people with cancer have been found to be comparable to age-matched peers.[^11^](#_ENREF_11)^,^ [^12^](#_ENREF_12) Specifically, one study exploring changes in TYA cancer patients (n=98, mean age=17.3 years) physical activity across the cancer continuum (pre-treatment, during treatment and post-treatment) found physical activity levels declined significantly during treatment and nearly one quarter of young people remained inactive post-treatment despite being active pre-treatment.[^13^](#_ENREF_13) Efforts are required to support TYA cancer survivors to make, and sustain, healthy lifestyle choices both during and after treatment.

At present, very little is understood about how best to deliver lifestyle information and behavior change support to TYA cancer survivors as much of the qualitative and quantitative evidence concerning the information needs and preferences of TYA cancer survivors has focused on late-effects management and psychosocial issues such as fertility, education, and relationships.[^8^](#_ENREF_8)^,^[^14-16^](#_ENREF_14) Ascertaining TYA cancer survivors’ specific needs and preferences regarding lifestyle information delivery is an important step in the development and design of health behavior interventions for young people with cancer. Such data gathered from a patient-centered perspective and used in the development of lifestyle intervention programs increases the likelihood that young people with cancer will engage with the lifestyle information being provided.[^17^](#_ENREF_17)

However, lifestyle information provision is passive and unlikely to prompt behavior change without the incorporation of behavior change techniques or an understanding of the underlying motives behind lifestyle change.[^18^](#_ENREF_18) Identifying barriers and facilitators of health behavior change among TYA cancer survivors is central to the design and development of interventions for this age group. Therefore, the aim of this study was to explore the lifestyle information needs of TYA cancer survivors and their preferences regarding health behavior change intervention delivery. In addition, this study aimed to explore barriers and facilitators to health behavior change among young people affected by cancer.

**Method:**

***Participants, Recruitment, and Ethical Approval***

Participants were TYA cancer survivors aged 13-25 years. Consistent with the National Cancer Institute definition of cancer survivor, any young person who had been diagnosed with cancer at any point within their lifetime was eligible to participate.[^19^](#_ENREF_19) Participants were recruited as part of a large-scale health and lifestyle survey being delivered to young people with cancer through University College Hospital, London and CLIC Sargent (a UK-based cancer charity supporting young people and their families). Surveys were anonymous, but participants who completed them had the option to express interest in being involved in a qualitative study focussing on the development of a lifestyle intervention for TYA cancer survivors. Young people had the option to choose the method of participation most convenient to them (a focus group or telephone interview). It was hoped that by proposing a combination of participation methods there would be increased interest. If a young person indicated interest in taking part in either a focus group or interview, they were sent an information sheet and consent form for the current study. All interviews and focus groups were conducted by the same person (GP) between July 2015 and January 2016. All participants provided informed written consent prior to the commencement of the interview or focus group. Young people received a £15 online shopping voucher for their participation. Ethical approval for this study was provided by University College London Research Ethics Committee reference: 6206/001 and London Hampstead Research Ethics Committee reference: 15/LO/0764.

***Interview topic guide***

The interviews and focus group followed the same semi-structured interview guide (provided as Document, Supplement Digital Content 1, http://links.lww.com/CN/A11) which focused on three main themes: i) what a healthy lifestyle means, ii) past experiences of receiving, asking and searching for lifestyle advice, and iii) preferences relating to lifestyle information delivery with regard to content, format and delivery. Participants were prompted when necessary and encouraged to share their thoughts and experiences openly.

***Analysis***

Qualitative data was transcribed verbatim and checked for accuracy during the familiarization phase of the six phase process of thematic analysis as outlined by Braun and Clarke.[^20^](#_ENREF_20) Following the generation of the initial list of emerging themes, several meetings between the study team (GP, AF, RB) were held until a single list of codes and themes was agreed upon. Each core theme and any unrelated sub-themes were then discussed with a TYA cancer expert (RH) for affirmation and identification of missing themes. An independent researcher (JH) then coded three of the interview transcripts to ensure each theme worked in relation to each coded extract. Any disagreements were resolved through discussion. All qualitative data analysis was carried out in NVivo version 11.0. To ensure transparency the final report and generation of results was guided by the consolidated criteria for reporting qualitative research (COREQ).[^21^](#_ENREF_21) Percentage agreement and cohens kappa were calculated to determine the inter-rater reliability of the analysis.

**Results**

From the original survey study (n=294), 93 young people left their contact details indicating interest in participating within this subsequent study. Of these, 13 young people responded to one of the three recruitment emails sent inviting them to take part in this study. In total, 10 young people took part in a telephone interview. Interviews typically lasted 30 minutes in duration (range: 19mins 41 seconds – 43 mins 16 seconds). A single focus group (n=3) was conducted in partnership with CLIC Sargent (Project Co-Funders) with members of the charities’ young people’s reference group using existing non-clinical meeting facilities within the charities London head office. The focus group lasted approximately 1.5 hours and was arranged at a time convenient to the group members.

The mean age of the sample was 22.9 years (range: 17-25 years) and the majority of participants were female (n=9, 70%). Mean age at diagnosis was 18.6 years. One participant was a TYA cancer survivor of a cancer diagnosed during childhood. Most participants (n=6, 46%) had been diagnosed with a hematological malignancy such as a leukemia or lymphoma (Table 1).

Three core themes emerged from the interviews and the single focus group: cancer as a catalyst to behavior change; factors influencing health behavior change; and health behavior information preferences. Figure 1 provides an overview of the thematic map generated within this study. The inter-rater agreement on emerging themes was high (mean weighted percentage agreement: 99.14%; mean weighted Kappa: 0.89).

***Cancer as a catalyst to lifestyle behavior change***

Young people indicated that their cancer diagnosis was often the underlying reason behind interest in lifestyle information and the catalyst behind any attempts to lead a healthier lifestyle. Leading a healthy lifestyle was seen by participants as a positive way of feeling better within themselves, regaining normality, regaining control of their health, and managing treatment related side-effects of cancer treatment including cancer recurrence. Participants also voiced an increased awareness of the importance of lifestyle for long-term health. This heightened awareness of health was often attributed to the impact of their cancer diagnosis on life outlook:

*‘I think being diagnosed with cancer you tend to be a bit more aware afterwards because you sort of don’t take your health for granted as much, I think.’ (Female, Aged 17, Diagnosed age 17, Wilms Tumor)*

With reference to smoking and tanning young people often indicated aversion to smoking *‘cos obviously its causes cancer…’* and increased awareness of the risks of tanning and sun exposure. Often young people indicated that their cancer and their treatment led them to be more conscious of their alcohol consumption but some admitted being *‘probably what you’d call a binge drinker’*. Two participants specifically indicated that they drink less now following their cancer diagnosis as they considered *‘more important things in life’* and *‘life too short to be unhealthy’.* Young people typically did not bring risk behaviors (smoking, drinking or tanning) up within discussion unless prompted.

***Factors Influencing Health Behavior Change***

It was evident that cognitive factors such as health beliefs, self-efficacy, and confidence often contributed to young people’s lifestyle behavior and engagement with health and lifestyle information:

*‘’Sometimes you have low self-esteem and you don’t want to… it’s not that the information isn’t engaging, it’s more how you feel in yourself’ (Female, aged 24, diagnosed age 21, Hodgkin lymphoma)*

Peer and social support were found to be the main facilitators of confidence and self-efficacy among young people. Young people often talked about the influence their immediate family and social network had upon their lifestyle both during and after treatment and emphasized the need for peer support to come from other young people of a similar age and position to them. Many young people looked to identify with other TYA cancer survivors and described wanting to know more about their lifestyles and what was *‘normal’* for them.

*‘You just kind of think, well, they understand, um, and I think it's a lot easier to get the information into your head when it's another person's story. Like, telling it rather than just someone whose job it is to write the information, it's coming from someone who's been through a similar situation….’ (Male, Aged 23, Diagnosed age 21, Hodgkin Lymphoma).*

All participants viewed education on the benefits of a healthy lifestyle and guidance on making lifestyle changes as important. Young people often reported that goals and progress monitoring would be useful motivational tools to encourage young people to sustain positive health behavior changes. Participants generally supported the notion that any changes should be made gradually in order to sustain health behavior change over-time and young people often acknowledged that a healthy lifestyle could be of benefit to everyone:

*‘I think a lot of people, yeah, they're disillusioned and think they can't exercise at all. But even a little bit is good for you. So it's just kind of making the best of what you've got, and being able to make a bit of time to keep active’ (Male, Aged 23, Diagnosed age 21, Hodgkin Lymphoma)*

Despite the high level of interest in leading a healthy lifestyle many young people described challenges they had faced in attempts to change their health behavior. Young people talked about the difficulty of having to navigate a *‘new body’* post treatment that is *‘not the same as what it was pre-treatment’*. Often treatment-related side-effects or late-effects were the main barrier to improving lifestyle behaviors, specifically diet or exercise related behaviors. Young people were often frustrated by treatment-related fluctuations in weight and failed attempts to maintain or reach a healthy body weight. Dietary habits and food preferences formed during treatment were often named as one of the biggest challenges to changing diet-related behaviors:

*‘I'd ate so much junk when I was on steroids that I just... it just became a habit, and I never broke it, really. So it's taken, like, six, seven years for me to break the unhealthy eating I was doing for two and a half years.’ (Female, Aged 25, Diagnosed age 15, Acute Lymphoblastic Leukemia).*

Specifically regarding physical activity, many participants shared that cancer and its associated treatment had led to a loss of confidence in their ability to exercise. One participant explained feeling self-conscious around other young people in public places such as local gyms because of cancer-related physical changes in his appearance such as *‘hair loss’* and *‘a fatter face cos of steroids…’*. In addition, young people often viewed themselves as different to other young people who had not had cancer:

*‘I’d never dreamt of going to the gym with… I don’t mean to say ordinary people, but people of good health because I would have felt like such an outsider.’ (Female, aged 24, diagnosed age 17, Hodgkin lymphoma)*

Geographical, financial, and time-related barriers to leading a healthy lifestyle were also mentioned by young people who indicated that travelling to support groups, paying for gyms, and preparing or cooking healthy meals were barriers to being active and eating healthily. Barriers to reducing alcohol consumption, quitting or abstaining from smoking, or being safe in the sun were often not discussed unless prompted. When prompted, young people were typically uncertain about the need to change these aspects of their lifestyle and cited lack of clear information as the primary barrier to change.

Although some young people did have a positive experience, most participants within this study reported a high level of dissatisfaction with the practical support and lifestyle information they had received in the past. Young people typically reported previously receiving very brief information about lifestyle from health professionals; often this advice was given within the context of treatment or during discussions about management of late-effects. Participants often reported that health professionals were not forthcoming with information about lifestyle and that either no information was given or that the information they were given was vague and out of context. One young person discussed an incident when a health professional was caught off guard by their request for specific information about sun safety:

*‘I did ask the doctor about, you know, “Was it okay to be in the sun?” ….I think it was a question that they weren’t really... I don't know if he’d been that, sort of, prepared for the question. He kind of was like, […] Oh, you’ll be okay [yeah] without sun cream if you’re outside for just a little while,’ which didn't really kind of answer... I’d quite like to have known, you know, what I... How... You know, can I go... Can I sit in the sun in the summer, for instance?’ (Female, Aged 22, Diagnosed aged 21, HLH/ Chronic Active EB Virus).*

Lack of information from health professionals often prompted young people to search elsewhere for information, most commonly online:

*‘So the first thing I done was went on the internet, googled it.’ (Female, Aged 24, Diagnosed age 17, Hodgkin Lymphoma)*

Young people’s level of satisfaction with the information they had found themselves varied. Some reported finding useful recipes and blogs from other cancer survivors whilst others reported struggling to find any age appropriate resources or any information relevant to their needs. Young people had strong views that there was not enough practical support available for young people with cancer that focused on TYA specific issues, specifically alcohol consumption:

*‘When you finished treatment you sort of feel a bit… not abandoned, but you are sort of on your own dealing with it yourself, trying to manage. You know, you feel like you are in charge of your own health again, which can be a bit of a responsibility. It would nice to be able to have someone […] giving you a bit of guidance’ (Female, Aged 24, Diagnosed Age 21, Bowel Cancer)*

*‘Yeah, because, um, I would like to know, you know, can I drink, will it affect me, you know, does it relate to like what kind of cancer I had, is too much… having too much bad for me? Probably anyway. But just, just in general like if, if I wanted to go out with my friends would I have a drink, because I don’t, I don’t know, I don’t know, if that makes sense, I don’t really know if I’m allowed to or not’ (Female, Aged 24, Diagnosed aged 18, CNS Tumor).*

***Health Behavior Information Preferences***

***Information Content***

Young people were interested in lifestyle information on a range of topics and suggested such information should be integrated with other information topics such as late-effects management and maintaining normalcy. Interest in specific information on weight maintenance was also common. Many participants expressed a specific interest in information about socializing, particularly drinking. Participants highlighted strongly that the content of lifestyle information for TYA cancer survivors should be relevant to the needs of young people with cancer and reflect the individual needs of survivors of specific cancers (Table 2).

***Resource Format***

Overall, young people were interested in information presented in a variety of formats that could be accessed depending upon their needs at any given time. Information delivered online or via mobile applications appealed to TYA cancer survivors as these formats were perceived as more accessible and appealing to young people (Table 3). However, several young people acknowledged that online information is only seen if it is being actively searched for. In such instances young people highlighted the need for information to be available in multiple formats including in the form of counselling from health professionals:

*‘I think online it tends to be… you only see it then if you go looking for it…. Like when I was diagnosed, my CLIC Sargent social worker came in and she just gave me loads of leaflets and just said, “Look through these,” and then I looked through them, whereas I wouldn’t have gone onto a website and looked at it myself, really’ (Male, Age 20, Diagnosed age 19, Acute Lymphoblastic Leukemia).*

Participants during discussion regarding the design and presentation of lifestyle information indicated that information should be concise, accessible, attractive and age-appropriate. Young people wanted lifestyle information that was easy to find and easy to navigate. Specific features such as designated topic sections, lists and interactive online features such as hyperlinked content and the ability to *‘pin’* or ‘*like*’ favorites appealed.

Young people expressed frustration at having to sift through *‘endless’* information resources in the past. Short articles or information presented in *‘chunks that you can read one by one’* were preferred. Participants also emphasized the need for lifestyle information resources to be designed specifically for *‘young people rather than children’*. Many of the participants emphasized the need for making sure both the content and format of health and lifestyle information was neither patronizing nor ‘*sugar-coated’*. Visual features such as use of color, infographics and imaginative page layout were suggested by young people as being key aspects of lifestyle information design and format.

***Timing and Delivery***

No consensus of when the best time to provide lifestyle information to young people with cancer was reached. Often young people felt that the correct time for lifestyle information delivery was dependent upon an individual’s frame of mind concerning their diagnosis, treatment status, and prognosis and that such information may be best delivered as and when a person is ‘ready’. Young people were often acutely aware of the risk-taking mind-set of young people and acknowledged that information delivered at the wrong time point can sometimes fall upon deaf ears. Participants stressed the importance of not being overwhelmed with information and that they were more receptive to information if advice was offered in a supportive and open manner. Young people also indicated that access to the information resources should be volitional and that young people should have the opportunity to be sign-posted to relevant information to take in during their own time:

*‘If somebody's kind of lectured you about it, and if you're kind of being told, "Oh yeah, go check this out, go check this out, go check this out," sometimes you just kind of think, "Oh, well, I don't really want to look at that." So I think, if they could be given a resource pack, maybe, to go and look into themselves…’ (Male, Age 23, Diagnosed aged 21, Hodgkin Lymphoma).*

Throughout all interviews and focus groups young people indicated a strong preference for information that was *‘sensible’* and *‘legitimate’* from reliable and trustworthy sources. Young people commonly cited well-known TYA cancer charities in the UK as the best sources of information and that they would prefer information endorsed by health professionals specializing in TYA care who *‘know what they are talking about’.*

**Discussion**

There is a need to provide young people who have had cancer with lifestyle information and health behavior change interventions to ameliorate their risks of chronic disease and cancer recurrence.[^22^](#_ENREF_22)^,^ [^23^](#_ENREF_23) Our findings highlight the need for readily available age-appropriate lifestyle information covering a wide-range of health topics for TYA cancer survivors. Such information should be incorporated into health behavior change interventions which support young people with cancer to make and sustain positive lifestyle changes.

In this study, young people often described their cancer diagnosis as the primary reason behind their interest in lifestyle information and engagement in health behavior change. Many young people reported an increased perception of personal health risk after their cancer diagnosis, and viewed adopting healthy lifestyle behaviors as a positive strategy to improve their health and well-being. There is potential that a cancer diagnosis may trigger an effective re-evaluation of health status among TYA cancer survivors and may potentially prime young people towards making healthier lifestyle choices. However, as with adult cancer survivors, it is unlikely that such spontaneous behavior change will occur, or be sustained over time, without intervention.[^24^](#_ENREF_24)^,^ [^25^](#_ENREF_25) Lifestyle information and behavior change delivery strategies should capitalize upon the potential *‘teachable moment’* when young people’s motivation to receive and act upon information or behavior change support is high.

Despite the high level of engagement with lifestyle-related topics, young people in this study often described numerous barriers to health behavior change. Specifically, many young people reported that cancer-related physical changes had negatively impacted their confidence and self-efficacy towards being active. This is consistent with previous reports indicating that the greatest psychosocial challenge faced by young people who have had cancer is the adjustment to physical and mental limitations resulting from their diagnosis and treatment^.^[^14^](#_ENREF_14) Content analysis of messages posted on an online forum for TYA cancer survivors confirm our findings that treatment related physical changes or physiological problems (such as gastrointestinal issues) promote anxiety and diminish self-efficacy among young people with cancer.[^26^](#_ENREF_26)

Conversely, social support emerged as a facilitator of both health behavior change and self-efficacy. Specifically, young people discussed the importance of social comparison and the value of knowing about the challenges other young people with a cancer faced when making lifestyle changes. Such findings support the overwhelming evidence concerning TYA cancer survivors’ need for social support both during and after treatment, and reflect the importance of behavioral modelling among adolescents and young adults.

Within this study, young people’s account of their experience and satisfaction of receiving and searching for information on physical activity, diet, drinking, smoking and sun safety was often negative, perhaps reflecting the lack of lifestyle information or behavior change resources available for TYA cancer survivors. This is concerning given the strong correlation between unmet information needs and poor health-related quality of life among TYA cancer survivors.[^27^](#_ENREF_27)^,^ [^28^](#_ENREF_28) Young people with cancer have previously reported lifestyle information as being *‘overlooked’* and specifically raised concerns about the impact of health professionals failing to address the consequences of alcohol consumption during treatment.[^29^](#_ENREF_29) A recent survey of TYA cancer survivors (n=216, mean age: 20 years) within the United Kingdom found the proportion of young people expressing interest in receiving advice on physical activity, diet, and weight management was greater than the proportion of young people who reported that they had actually received advice on these topics suggesting some TYA cancer survivors have un-met information needs in these areas.[^30^](#_ENREF_30) Addressing the specific lifestyle information needs of young people with cancer is important as information provision is a core aspect of supportive cancer care. [^31^](#_ENREF_31)

Young people in this study were interested in age-appropriate lifestyle information specific to their needs as TYA cancer survivors. A desire for lifestyle information that includes reference to side-effects or late-effects management was also common. This is reflective of previous reports detailing young people’s high level of interest in discussing health behavior during late effects consultations.[^9^](#_ENREF_9) In addition, consistent with previous research exploring intervention design and delivery preferences, TYA cancer survivors within this study specified that lifestyle information and health behavior change interventions should be readily available and continually accessible.[^32^](#_ENREF_32)^,^ [^33^](#_ENREF_33) Young people within this study also specified that lifestyle information should be available in multiple formats to suit individual preferences. Participants discussed the explicit advantages of lifestyle information available online, highlighting the accessibility of this format of information and intervention delivery. However, despite young people indicating a strong preference for lifestyle information and health behavior interventions to be made available and delivered online, young people acknowledged that information delivered via such mediums lack the personal support from health professionals, immediate family, and social networks, which they like. This is reflective of parent and professional opinion expressed within previous research exploring the user requirements and considerations of web-based self-management for TYA cancer survivors: parents and health professionals both stressed the importance of online information resources not replacing face-to-face consultations and interactions with either peers or health professionals.[^34^](#_ENREF_34)

No consensus as to when the most appropriate time to introduce lifestyle information to young people with cancer was reached. Consistent with the thoughts of TYA cancer specialists, participants within this study typically felt that the timing and delivery of lifestyle information should take into account the individual needs of the young person being given advice*.* However, it was generally agreed that although health behavior change may not occur immediately it would be beneficial to introduce TYA cancer survivors to the concept of lifestyle change as early as possible in the cancer care pathway. Such early introductions to lifestyle and health behavior change information may prevent the accumulation of bad habits, trigger behavior change among some young people, and prime those who are not yet ready for lifestyle change to at least be thinking about this issue.

This study has a number of strengths and limitations. A major strength of this study is the inclusion of young people’s perspectives on drinking, smoking and sun safety. The relevance of these behaviors to TYA cancer survivors is often overlooked within studies which typically address diet and physical activity. Moreover, this study provides qualitative insight into some of the previously reported correlates of health behavior among TYA cancer survivors.[^35^](#_ENREF_35)^,^[^36^](#_ENREF_36) Such information is invaluable to the development and design of health behavior interventions for TYA cancer survivors. We aimed to obtain a breadth of opinions from a wide range of TYA cancer survivors, however, very few young people answered our call for participants despite having indicated an interest in taking part during a previous study. A mixed method qualitative approach was taken and incentives were introduced to this study in order to overcome these recruitment barriers; these decisions were based upon previous reports detailing the difficulties typically faced within TYA cancer survivorship research.[^37^](#_ENREF_37)^,^ [^38^](#_ENREF_38) Although there were no differences between the qualitative data generated from the interviews and focus group, social barriers during the focus group may have reduced the likelihood of an individual sharing an experience or idea. It would also be reasonable to suggest that the young people who took part within this study were engaged with health and lifestyle information and as a result the possibility of response bias is high. Further research to clarify the lifestyle information needs and health behavior interventions preferences of TYA cancer survivors with low levels of engagement is required.

**Implications for Practice**

Our findings highlight the need for readily available age-appropriate lifestyle information for young people with cancer covering a wide-range of health topics. This is supported by recent quantitative data indicating that 71% of TYA cancer survivors would take up the offer of lifestyle behavior information and support if given the opportunity.[^30^](#_ENREF_30) Given the correlation between health beliefs and behavioral intention among TYA cancer survivors health behavior change interventions developed and designed specifically for TYA cancer survivors should facilitate self-efficacy through social support, goal setting, and behavior tracking. Nurses and health professionals working with TYA cancer survivors should address young people’s lifestyle information needs throughout the cancer care pathway and support young people to foster the confidence to make, and sustain, positive lifestyle behavior changes. Within this context peer-to-peer support from another young person with cancer may also be hugely beneficial to TYA cancer survivors struggling to lead a healthy lifestyle following their cancer diagnosis. Greater insight into specific correlates of health behavior and the differences between health protective behaviors such as physical activity and diet and risk behaviors such as drinking, smoking and tanning are required. The development and design of health behavior interventions for TYA cancer survivors must also consider the perspective of health professionals working within this field.

**Conclusions**

The findings from this study demonstrate that lifestyle information needs of TYA cancer survivors are currently unmet and that tailored approaches to health behavior change among this group of cancer survivors are desired. It is evident that health behavior promotion among TYA cancer survivors is complex and lifestyle information regarding physical activity, diet, drinking, smoking and sun safety should be delivered to young people and must consider the disease-specific barriers young people with cancer encounter when making lifestyle related changes.

**References**

1. O’Hara C, Moran A, Whelan J, et al. Trends in survival for teenagers and young adults with cancer in the UK 1992–2006. *Eur. J. Cancer.* 2015; 51(14): 2039-2048.

2. Hudson M, Ness K, Gurney J, et al. Clinical ascertainment of health outcomes among adults treated for childhood cancer. *JAMA*. 2013; 309(22): 2371-2381.

3. Robison LL, Hudson MM. Survivors of childhood and adolescent cancer: life-long risks and responsibilities. *Nat Rev Cancer*. 2014; 14(1): 61-70.

4. Spector DJ, Noonan D, Mayer D, et al. Are lifestyle behavioral factors associated with health-related quality of life in long-term survivors of non-Hodgkin lymphoma? *Cancer.* 2015; 121(18): 3343-3351.

5. Siegel R, Lockhart M, Kist C. Reducing lifestyle risk in Childhood Cancer Survivors. *Acta paediatrica.* 2015; 104(10): 969-970.

6. Tonorezos ES, Robien K, Eshelman-Kent, et al. Contribution of diet and physical activity to metabolic parameters among survivors of childhood leukemia. *Cancer causes control*. 2013; 24(2): 313-321.

7. Jones LW, Liu Q, Armstrong GT, et al. Exercise and Risk of Major Cardiovascular Events in Adult Survivors of Childhood Hodgkin Lymphoma: A Report From the Childhood Cancer Survivor Study. *J. Clin. Oncol.* 2014; 32(32): 3643-3650.

8. Nass SJ, Beaupin LK, Demark-Wahnefried W, et al. Identifying and Addressing the Needs of Adolescents and Young Adults With Cancer: Summary of an Institute of Medicine Workshop. *The Oncologist.* 2015; 20(2): 186-195.

9. Michel G, Greenfield D, Absolom K, et al. Follow-up care after childhood cancer: Survivors’ expectations and preferences for care. *Eur. J. Cancer.* 2009; 45(9): 1616-1623.

10. Murnane A, Gough K, Thompson K, et al. Adolescents and young adult cancer survivors: exercise habits, quality of life and physical activity preferences. *Supp Care Cancer.* 2015; 23(2): 501-510.

11. Marjerrison S, Hendershot E, Empringham B, et al. Smoking, Binge Drinking, and Drug Use Among Childhood Cancer Survivors: A Meta-Analysis. *Pediatr Blood Cancer.* 2016; (63) 1254-1263

12. Badr H, Chandra J, Paxton R, et al. Health-related quality of life, lifestyle behaviors, and intervention preferences of survivors of childhood cancer. *J Cancer Surviv*. 2013; 7(4): 523-34.

13. Keats MR, Culos-Reed N, Courneya K, McBride M. An examination of physical activity behaviors in a sample of adolescent cancer survivors. *J Pediatr Oncol Nurs.* 2006; 23(3) 135-142

14. Barakat L, Galtieri L, Szalda D, et al. Assessing the psychosocial needs and program preferences of adolescents and young adults with cancer. *Supp Care Cancer.* 2016; 24(2): 823-832.

15. Morgan S, Davies S, Palmer S, Plaster M. Sex, drugs, and rock 'n' roll: caring for adolescents and young adults with cancer. *J Clin Oncol.* 2010. 28(32): 4825-4830.

16. Zebrack B. Information and service needs for young adult cancer survivors. *Supp Care Cancer*. 2008. 17(4): 349-357.

17. Zebrack B. Patient-centered research to inform patient-centered care for adolescents and young adults (AYAs) with cancer. *Cancer.* 2014; 120(15): 2227-2229.

18. Michie S, Van Stralen MM, West R. The behaviour change wheel: A new method for characterising and designing behaviour change interventions. *Implement Sci.* 2011; 6:42.

19. National Cancer Institute. Definition of Survivor - NCI Dictionary Cancer Terms - National Cancer Institute website. http://www.cancer.gov/publications/dictionaries/cancer-terms?cdrid=450125. Last Accessed August 12 2016.

20. Braun, V. and V. Clarke (2006). Using thematic analysis in psychology. Qualitative Research in Psychology 3(2): 77-101.

21. Tong A, Sainsbury P, Craig J. Consolidated criteria for reporting qualitative research (COREQ): a 32-item checklist for interviews and focus groups. *Int J Qual Health Care.* 2007; 19(6): 349-357.

22. Barnes MJ, Demark-Wahnefried W. Importance of balanced diet and physical activity during and after cancer treatment in adolescent patients. *Clin Oncol Adolesc and Young Adults*. 2014; 4:13.

23. Carretier J, Boyle H, Duval H, et al. A Review of Health Behaviors in Childhood and Adolescent Cancer Survivors: Toward Prevention of Second Primary Cancer. *J Adolesc Young Adult Oncol.* 2015; 5(2)78-90

24. Demark-Wahnefried W, Aziz NM, Rowland JH, Pinto BM. Riding the crest of the teachable moment: promoting long-term health after the diagnosis of cancer. *J Clin Oncol.* 2005; 23(24): 5814-30.

25. Williams K, Steptoe A, Wardle J. Is a cancer diagnosis a trigger for health behaviour change? Findings from a prospective, population-based study. *Br J Cancer.* 2013; 108(11): 2407-2412.

26. Love B, Moskowitz M, Crook B, et al. Defining adolescent and young adult (AYA) exercise and nutrition needs: Concerns communicated in an online cancer support community. *Patient Educ Couns.* 2013; 92(1): 130-133.

27. Gupta AA, Edelstein K, Albert-Green A, D’Agostino N. Assessing information and service needs of young adults with cancer at a single institution: the importance of information on cancer diagnosis, fertility preservation, diet, and exercise. *Support Care Cancer*. 2013. 21(9): 2477-84.

28. Smith, AW, Parsons HM, Kent EE, et al. Unmet Support Service Needs and Health-Related Quality of Life among Adolescents and Young Adults with Cancer: The AYA HOPE Study. *Front Oncol.* 2013; 3: 75.

29. Fern LA, Lewandowski JA, Coxon KM, Whelan J. Available, accessible, aware, appropriate, and acceptable: a strategy to improve participation of teenagers and young adults in cancer trials. *The Lancet Oncology.* 2014. 15(8): e341-e350

30. Pugh, G., et al., (2017) The provision of health behaviour information to teenage and young adult cancer survivors Journal of Adolescent and Young Adult Oncology. Online Ahead of Print.

31. Taylor RM, Pearce S, Gibson F, Fern L, Whelan J. Developing a conceptual model of teenage and young adult experiences of cancer through meta-synthesis. *Int J Nurs Stud.* 2013. 50(6): 832-846.

32. Rabin C, Simpson N, Morrow K, Pinto B. Intervention format and delivery preferences among young adult cancer survivors. *Int J Behav Med.* 2013. 20(2): 304-310.

33. Belanger LJ, Plotnikoff R, Clark A, Courneya KS. A survey of physical activity programming and counseling preferences in young-adult cancer survivors. *Cancer Nurs.* 2012. 35(1): 48-54.

34. Moody L, Turner A, Osmond J, Hooker L, et al. Web-based self-management for young cancer survivors: consideration of user requirements and barriers to implementation. J Cancer Surviv. 2015. 9(2): 188-200.

35. Vuotto S, Procidano M, Annunziato R. Understanding the Health Behaviors of Survivors of Childhood and Young-Adult Cancer: Preliminary Analysis and Model Development. *Children.* 2015. 2(2): 174.

36. Gilliam MB, Schwebel DC. Physical Activity in Child and Adolescent Cancer Survivors: A Review. *Health Psychology Review*. 2013. 7(1): 92-110.

37. Rabin C, Horowitz S, Marcus B. Recruiting Young Adult Cancer Survivors for Behavioral Research. *J Clin Psychol Med Settings.* 2013. 20(1): 33-36.

38. Hendricks-Ferguson, V.L., et al., Recruitment Strategies and Rates of a Multi-Site Behavioral Intervention for Adolescents and Young Adults With Cancer. *Journal of Pediatric Health Care.* 2013. 27(6): 434-442.

**Table and Figure Legend**

Table 1. Participant Characteristics

Table 2. Lifestyle Intervention Preferences: Content

Table 3. Lifestyle Intervention Preferences: Format

Figure. Thematic map of the lifestyle information and intervention preferences of teenage and young adult cancer survivors.
