# Supplementary material for: The Lifestyle Information and Intervention Preferences of Teenage and Young Adult Cancer Survivors: A Qualitative Study
Source: Cancer Nurs. 2017 Jun 15;41(5):389–98. doi: 10.1097/NCC.0000000000000508 (PMC6259678; doi:10.1097/NCC.0000000000000508)
Supplement: SUPPLEMENTARY MATERIAL [file ncc-41-389-s003.docx]

Department of Epidemiology

& Public Health

Health Behaviour RESEARCH CENTRE

**Lifestyle Advice for young survivors: focus groups & interviews**

**Focus Group and Interview Schedule**

**Aim: To explore Teenage and Young Adult Cancer Survivors views about lifestyle and how they would like to receive such advice.**

**Introductions & House-keeping**

- Introduce self and the aim of the group meeting.
- Check the length of the session is okay for everyone.
- Highlight fire exits and where the bathrooms are.
- Remind participants that discussion is confidential and that views shared within the group should not be discussed outside the room.
- Remind participants to say their name before they speak so we can identify who is talking and when.
- Remind participants that if they wish to leave at any time they are free to do so.
- Check everyone is okay and comfortable.

|  |  | Prompts |
| --- | --- | --- |
| **Participant Introductions** | *’Can you please tell us your name, age, your cancer diagnosis and what treatments you are currently or have previously received’* | - When diagnosed - Type of cancer - Treatment - Recovery |
| **Lifestyle** | *‘What is a healthy lifestyle to you?’* | - Healthy eating - Physical Activity - Alcohol & Smoking - Sun Safety |
|  | *‘Have any of you ever tried to change your health behaviour since your diagnosis with cancer - What changes did you make?* | - Where you successful? - Did you feel supported to make these changes? |
|  | *What challenges have you faced when you have tried to change your lifestyle?* | - Barriers   - Lack of support?   - Time?   - Health? |
|  | *‘Have any of you ever been given advice about lifestyle from a health professional, what did they tell you?* | - Healthy eating - Physical Activity - Alcohol & Smoking - Sun Safety |
|  | *‘What kind of information would you like to receive about lifestyle?’* | - Healthy eating - Physical Activity - Alcohol & Smoking - Sun Safety |
| **Sources of information** | *‘Has anyone ever tried to go and find information out for themselves - Where did you look, what did you find?’* | - Online - Social workers - App - Good experience/ bad experience |
|  | *Did anyone assist you in finding out this information?* | - Parent - Sibling/ Friend - Social Worker |
| **Delivery** | *‘When do you think lifestyle advice should be given to young people who have had a cancer diagnosis’* | - Before treatment, post treatment, at late effects clinic |
|  | *‘Who would you want to talk to about this kind of information?’* | - Social Worker - Doctor - Nurse - Parent - Trusted Friend |
|  | *How do you think information should be given out?* | - Online - Internet - Leaflet - Group counselling - App |
|  | *Where should this information be made available?* | - Internet (home) - in clinics (hospital) - by social workers (in the community) |
|  | *What do you think about setting goals or keeping a log?* | - Suggest other BCT - Self-help manual? |
|  | *How should information look?* | - Small bitesize pieces of information? - Lists? - Facts? - Instructions? - Pictures? |
|  | *How would you feel about participating in a group programme?* |  |
|  | *What are your thoughts about an app to support behaviour change?* | - Good/ Bad? - Explore why? |
|  | *Do you have any advice for us as we develop and design a lifestyle intervention for young people who have had a cancer diagnosis.* |  |
| **Conclusion** | *Anything else?* |  |
